# Population Pharmacokinetics of Vancomycin in Thai Patients

**DOI:** 10.1100/2012/762649

**Published:** 2012-04-01

**Authors:** Tunggul Adi Purwonugroho, Suvatna Chulavatnatol, Yupaporn Preechagoon, Busba Chindavijak, Kumthorn Malathum, Pakwan Bunuparadah

**Affiliations:** ^1^Faculty of Pharmacy, Mahidol University, Rajathevi, Bangkok 10400, Thailand; ^2^Department of Pharmacy, Faculty of Medicine and Health Sciences, Jenderal Soedirman University, Purwokerto 53123, Indonesia; ^3^Faculty of Pharmaceutical Sciences, Khon Kaen University, Khon Kaen 40002, Thailand; ^4^Faculty of Medicine, Ramathibodi Hospital, Mahidol University, Bangkok 10400, Thailand

## Abstract

Population pharmacokinetics of vancomycin in Thai adult patients was determined by non-linear mixed-effects approach using 319 vancomycin serum concentrations from 212 patients. The data were best fitted by a two-compartment model and it was used to examine the effect of patient characteristics on the vancomycin pharmacokinetics. In the final model, there was a linear relationship between vancomycin clearance, CL (L/h), and creatinine clearance calculated by Cockcroft-Gault equation, CL_Cr_ (mL/min): CL  =0.044  ×  CL_Cr_. Meanwhile, volume of central compartment, *V*_1_ (L), was linearly related with the age (years old): *V*_1_ = 0.542  × Age. Intercompartment clearance (*Q*) and volume of peripheral compartment (*V*_2_) was 6.95 L/h and 44.2 L, respectively. The interindividual variability for CL, *V*_1_, *Q*, and *V*_2_ was 35.78, 20.93, 39.50, and 57.27%, respectively. Whereas, the intraindividual variability was 4.51 mg/L. Final model then was applied to predict serum vancomycin concentrations on validation group. Predictive performance revealed a bias of −1.43 mg/L (95% CI: −5.82–2.99) and a precision of 12.2 mg/L (95% CI: −1.60–26.16). In conclusion, population pharmacokinetic of vancomycin in Thai adult patients was developed. The model could be used to create vancomycin dosage regimen in the type of patient similar with the present study.

## 1. Introduction

Vancomycin, an antibiotic with glycopeptide structure, is one of a few antibiotics available to treat patients infected with methicillin-resistant *Staphylococcus aureus *and methicillin-resistant coagulase-negative staphylococcal species. Its' pharmacokinetics could be changed by the patient conditions such as renal function [1], age [2], body weight [3], critical illness [4], type of dialysis [5], and type of infection [6]. Complexity on using this drug was also raised by the emergence of vancomycin-intermediate susceptible and vancomycin-resistant pathogens [7]. 

In clinical practice, approaches to dosing of vancomycin include empirical dosing [8] and dosing by nomograms [9, 10]. However, these methods were developed based on pharmacokinetic parameters derived from a group of Western population who may have different parameter values from Thai patients. Application of vancomycin nomograms or dosing methods developed from non-Thai patients in Thai patients might pose to undesired outcome due to the facts of narrow therapeutic index and large variability exhibited by this drug. Present study was performed to elucidate the pharmacokinetic parameters and factors that may affect vancomycin pharmacokinetics in Thai population.

## 2. Methods

Patients with the age of more than 18 years old in any ward at Ramathibodi Hospital, Mahidol University, Bangkok, Thailand who received vancomycin with complete data regarding dosage regimens, serum drug concentration, and precise timing of dose administration and blood sampling over the entire course of vancomycin therapy during January, 2007 till June, 2010 were included. The study protocol was approved by Human Research Ethics Committee of Faculty of Medicine, Ramathibodi Hospital, Mahidol University.

Patients were divided randomly into two groups. The first group, as a modeling group, was used to build the population pharmacokinetic model. After the population model had been defined, it was used to predict a serum vancomycin concentration in the second group, a validation group. Categorical data were analyzed by *χ* ^2^ test. Continuous data were evaluated for normality of distribution by Kolmogorov-Smirnov test. Normally distributed continuous data were analyzed by Student's *t*-test or Mann-Whitney *U* test if the data were not normally distributed. Differences between these groups were considered to reach statistical significance when *P* < 0.05.

Measurement of serum vancomycin concentrations were conducted as part of therapeutic drug monitoring activity in the hospital using the fluorescence polarization immunoassay (FPIA) method with Axsym system. The assay sensitivity limit was reported to be 2.00 mg/L. Furthermore, the coefficient of variation was 4.26%, 2.94%, and 4.06% at concentrations of 7.0, 35.0, and 75.0 mg/L, respectively (package insert, Axsym system, Abbot Laboratories, Abbot Park, Ill, USA).

### 2.1. Model Building

Pharmacokinetics (PK) modeling were developed using NONMEM (nonlinear mixed-effects modeling) software package version VII (Project Group, University of California, San Francisco, Calif, USA). On the basis of individual data set, a population value of pharmacokinetic parameters were determined, as fixed-effect parameters. Additionally, interindividual variability and the intraindividual variability were estimated as random effects. 

To determine the most suitable compartmental model, data from vancomycin concentrations versus time were fitted to both one- and two-compartment models, with first-order elimination, specified to NONMEM by ADVAN1-TRANS2 or ADVAN3-TRANS4 subroutines, respectively. The fixed-effect PK parameters estimated directly with these model specifications were total body clearance (CL) and volume of distribution (*V*) for the one-compartment model. For the two-compartment model, CL, volume of distribution of the central compartment (*V*_1_), intercompartment clearance (*Q*), and volume of distribution of the peripheral compartment (*V*_2_) were estimated. Additive, proportional, and exponential-error models were tested to describe interindividual and intraindividual variability. 

After basic model was determined, each of covariate was included one by one to basic model to get covariate that significantly improved the ability of the model to predict the observed concentration-time profile (preliminary screening phase). The covariates of patients were tested for their influences on vancomycin pharmacokinetic parameters as follows: age, gender, weight, serum creatinine concentration, blood urea nitrogen (BUN), creatinine clearance (using Cockcroft-Gault/C-G equation [11] and Modification of Diet in Renal Disease/MDRD4 equation [12]), serum albumin concentration, total and direct bilirubin level, aspartate transaminase (AST) and alanine transaminase (ALT) level, presence or absence of ascites, and concomitant drugs (aminoglycosides, amphotericin B, nonsteroidal anti-inflammatory drugs (NSAIDs), dopamine, dobutamine, adrenaline/noradrenaline, and furosemide). Then, the significant covariates were added cumulatively (forward stepwise fashion) to the model in the order of their contribution to the reduction in the objective function value (OFV) in the preliminary analysis until there was no further reduction in OFV. Finally, back elimination was conducted to eliminate any unnecessary covariates from the full regression model in descending order of their contribution to the change in OFV. 

The best compartment model (one versus two compartment), error model (additive versus proportional versus exponential error models), and the retention of covariate(s) in the model were determined by statistical significance of the model and evaluated by the likelihood ratio test using the minimum value of the OFV (−2 log likelihood difference), produced by the NONMEM program. Changes in the OFV >6.63 and >9.21 were determined to be significant on the basis of a *χ* ^2^ distribution with 1 degree of freedom (*χ* ^2^, *df* = 1, *P* < 0.01) and 2 degree of freedom (*χ* ^2^, *df* = 2, *P* < 0.01), respectively.

Other diagnostic criteria were a reduction in unexplained interindividual variability for the associated PK parameter and an improvement in the graphic diagnostic model. Graphics were obtained by plotted observed versus predictive concentrations and observed versus weighted residuals (predicted minus observed concentration and weighted by standard deviation).

### 2.2. Predictive Performance

Population-based parameters developed from model group were used to predict a value of vancomycin concentration in the validation group. This was performed by holding constant the values of the fixed and random effects parameters of the final population model and running the POSTHOC option in the $ESTIMATION procedure without allowing NONMEM to iterate. The mean error (ME) was used to measure bias, while precision was determined by calculating the mean squared error (MSE) and the root mean squared error (RMSE). These values with 95% confidence interval for the true mean were estimated by equation described by Sheiner and Beal [13]. Graphics were additionally used to determine the deviations between measured and predicted concentration.

## 3. Results

Using inclusion criteria described above, 268 patients in total were included. After randomization, 228 patients belonged to modeling group and 40 patients to the validation group. However, in further steps, the numbers of data were reduced into 212 patients and 34 patients in modeling and validation group, respectively. In modeling group, the number of patients was reduced in final model, since the covariate model building processes relied on the availability of particular covariate within the patients. Meanwhile, the reduced number of patients in the validation group occurred, because some patients were rejected, since they could not produce the parameters when POSTHOC option was applied. Then, this final figure (Table 1) was used in further analysis. 

**Figure 1:**
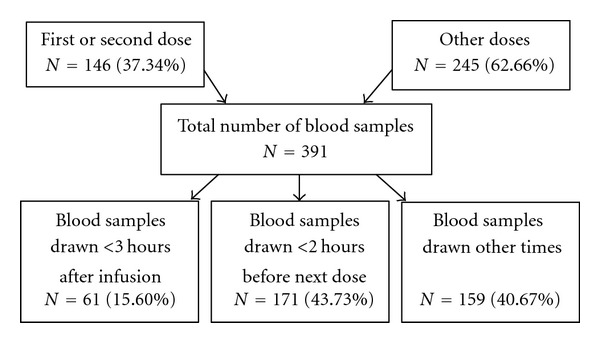
Number of vancomycin serum concentrations regarding to blood sampling time in modeling group.

The total number of serum concentrations in modeling group was 391 samples accounted as 1.84 concentrations per patient on average (ranging from 1 to 8 samples/patient). The dosage regimen was varied between patients and within patient. The serum concentrations in regard to blood sampling times (peak, trough, and other) also varied as described in Figure 1. 

In the basic model building step (using 432 concentrations from 228 patients), compartment model was compared between one and two compartment. The results preferred two-compartment model as better model to describe the present population (the decrease in OFV from one-compartment to two-compartment model was 154.346; *χ* ^2^, *df* = 2, *P* < 0.01). The error models to describe inter- and intraindividual variability were influenced by the nature of data [14]. In the present study, proportional error model was best described for interindividual variability, since there was a large degree of variability in the true parameters. On the other hand, an additive error model was chosen for intraindividual variability, since the concentrations were within the narrow range of distribution (89.51% of concentrations were on range of 2–30 mg/L).

In the covariate preliminary screening step, covariates were divided into three groups on the basis of the similarity of number of patients who had these covariates. This approach was implemented, since major diagnostic criteria, OFV produced by NONMEM, were influenced by the amount of data. The less the number of data, the value was expected to be reduced. In other words, decreasing in OFV did not always mean the model was a better model when the number of data was different. 

The first group with 228 patients and 432 levels was tested for covariates of age, gender, weight, serum creatinine concentration, and creatinine clearance (using C-G equation and MDRD4 equation). For vancomycin clearance, three covariates which reduced OFV more than 6.63 were CL_Cr_ by C-G equation, CL_Cr_ by MDRD4 equation, and serum creatinine concentration. Creatinine clearance from both equations also became significant covariates for volume of peripheral compartment. Volume of central compartment was significantly affected by creatinine clearance by C-G equation. Another significant covariate was age, which affected volume of central compartment. Other covariates (BUN, serum albumin concentration, total and direct bilirubin level, AST and ALT level, and presence or absence of ascites) were tested in 207 patients, using 384 levels. It revealed that ascites became a significant covariate for volume of central compartment and volume of peripheral compartment and BUN for vancomycin clearance. Meanwhile, concomitant drugs (aminoglycosides, amphotericin B, NSAIDs, dopamine, dobutamine, adrenaline/noradrenaline, and furosemide) were analysed separately in 216 individuals with 400 vancomycin levels. Drugs were also combined and then tested as two groups, which were drugs with nephrotoxic properties (aminoglycosides, amphotericin B, NSAIDs, and furosemide) and drugs with haemodynamic effect (dopamine, dobutamine, adrenaline/noradrenaline, and furosemide). As a result, there were no single or combination drugs that significantly affected the parameters in the model.

Before forward stepwise analysis was implemented, those significant covariates were regrouped and combined with as many as patients who had data for those covariates. Eventually, 212 patients were chosen and checked for their contribution to the model, similar manner with process in preliminary screening phase. Although serum creatinine generated a significant decrease in OFV, it was omitted in this step since the contribution was much lesser than CL_cr_ by C-G equation and CL_Cr_ by MDRD4 equation. 

In forward step fashion, a covariate with the highest influence was inserted to the model, followed by other less significant covariates. When such covariate in particular parameter significantly reduced OFV, this covariate would be retained in the model for further covariate analysis, and vice versa. However, creatinine clearance by MDRD4 equation and BUN were excluded in this step, since they had similar physiologic basis with creatinine clearance by C-G equation. Additionally, the C-G equation was more widely used in clinical practice than MDRD4 equation to predict the renal function of the patients. In case of BUN, although the OFV was decreased significantly from basic model, the value was much higher than those from C-G and MDRD4 equation, indicating the less significant contribution to the model. 

**Figure 2:**
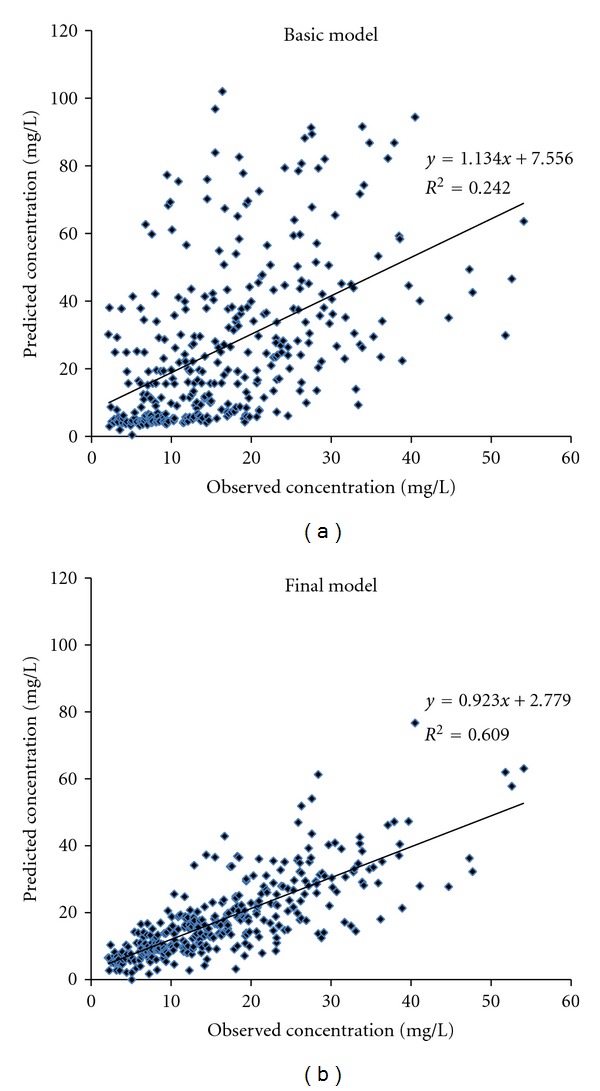
Scatter plot of observed against predicted concentrations in modeling group obtained from basic model (two-compartment model, without covariate) and final model (two-compartment model, with covariates).

**Figure 3:**
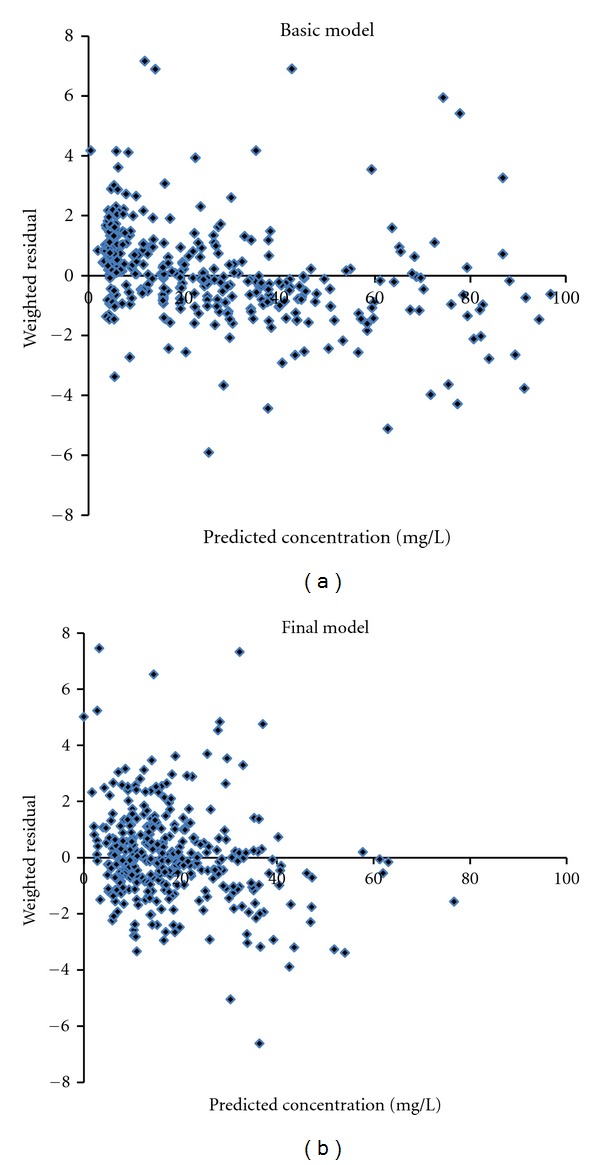
Scatter plot of predicted concentrations against weighted residuals in modeling group obtained from basic model (two-compartment model, without covariate) and final model (two-compartment model, with covariates).

As the result, in forward stepwise, the cumulative inclusion of covariates creatinine clearance by C-G equation on CL and age on *V*_1_ reduced OFV by more than 6.63 at each addition. These were confirmed by back elimination step. Accordingly, the model was considered as final model of vancomycin population pharmacokinetics in Thai patients (Table 2). Further inspection in scatter plot verified that the final model was better than basic model (two-compartment model without covariate), as the plots were distributed closer to the trend-line (Figure 2) and zero line (Figure 3), respectively. 

**Figure 4:**
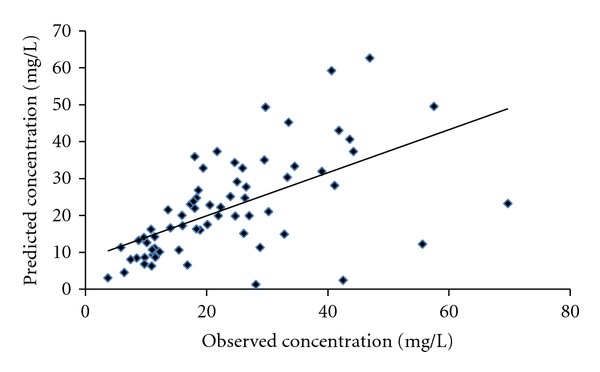
Scatter plot of observed against predicted concentrations of validation group.

**Figure 5:**
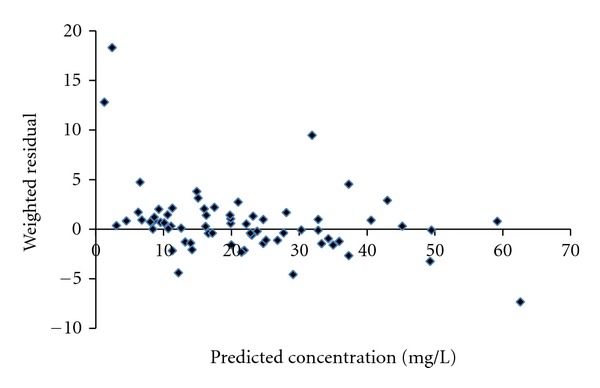
Scatter plot of predicted concentrations against weighted residuals of validation group.

Sixty-eight vancomycin concentrations from 34 patients from validation group were used to validate the final model. These observed concentrations were compared with predicted concentrations calculated from parameters obtained from the modeling group to determine the predictive performance. The mean prediction error (ME) as bias measurement was −1.43 mg/L with 95% confidence interval (95% CI) of −5.82–2.99 mg/L. Meanwhile, precise measurement as expressed by root mean squared prediction error (RMSE) with 95% CI was 12.28 mg/L (−1.60–26.16 mg/L). Two scatter plots (Figures 4 and 5) were taken to demonstrate the deviations of predictive and observed concentrations pairs.

## 4. Discussion

The present study used a population approach to determine the pharmacokinetic parameters of vancomycin in Thai patients along with their inter- and intraindividual variabilities. Vancomycin was a chosen drug in this study due to its large pharmacokinetic variabilities. Moreover, the studies about pharmacokinetic parameters of vancomycin in Thai population were not available. 

Since the renal pathway is the major one for vancomycin excretion, this is not surprising to elicit creatinine clearance as significant factor for vancomycin clearance. Additionally, the slope of vancomycin clearance-creatinine clearance relationship in the present study (0.044) was very close with the study in Japanese population (0.048) [15]. The value of vancomycin clearance using mean value of creatinine clearance in our study was 1.56 L/h. This value was lower approximately two times than other previous population studies using similar two-compartment model [4, 15, 16]. The difference may be due to the lower creatinine clearance in present population, which accounted about 50% lower than those populations. 

The finding of increasing *V*_1_ in accordance to increasing age in the present study disagreed with result from other study in healthy populations [17]. In that study, *V*_1_ of six men with a mean age of 23 years old was not different from that of older six men with a mean age of 68 years old. Differences of study population might contribute to this dissimilarity. Age influence on *V*_1_ does not seem to have direct physiological basis. Instead, this might be due to age being a surrogate of illness severity or other nonobserved covariates. In this study, the mean of intercompartmental clearance (*Q*) was slightly lower than previous studies [4, 15, 16]. It might be caused by decrease in cardiac output due to their underlying disease of elderly patients. Decreasing the *Q* resulted in a more rapid decrease in plasma concentrations following long infusion (like in the case of vancomycin administration). Therefore, the volume of central compartment might be increased as a net result.

In the present study, *V*_1_ and *V*_2_ did not associate with patient's body weight. The narrow range of body weight on majority patients (86% of patients' body weights were ranged from 40–70 kg) might not vary enough to become a significant covariate in this population. Additionally, the volume of central compartment (*V*_1_) was significantly related to age. These values (*V*_1_: 0.63 L/kg and *V*_2_: 0.77 L/kg) seemed to be larger than previously reported values, particularly in noncritically ill patients. Hurst et al. found the *V*_1_ value of 0.14 L/kg in cardiac outpatients who required a single dose of vancomycin for prophylaxis prior to dental procedure [18]. Meanwhile, in the study that included patients with various degree of renal dysfunction, *V*_1_ was estimated as 0.21–0.24 L/kg [19]. However, when compared with study in critically ill patients, the values of volume of distribution in the current study seemed to be smaller, especially in the value of *V*_2_ and volume of distribution at steady state or VD_ss_ (VD_ss_ = *V*_1_ + *V*_2_). In ICU patients, the VD_ss_ value of 1.73 L/kg was found in the study conducted by Llopis-Salvia and Jiménez-Torres [4]. Using similar pattern of patients, but with one compartment model, Del Mar Fernandez De Gatta Gracia et al. got VD of 1.68 L/kg [20]. Both are larger than VD_ss_ estimated from our study (1.39 L/kg). In the case of critically ill patients, the increased volume of distribution was attributed to the sepsis-induced interstitial space fluid [21]. Type of patients included in the current study as heterogenous patients (about one third of patients belonged to ICU patients) might explain why the value of volume of distribution fell in between those of noncritically ill and critically ill patients' values.

Insertion of significant covariates to the basic model decreased the magnitude of interindividual variability, particularly on parameter of clearance. The %CV of 111.80% in basic model was decreased to 35.78% after creatinine clearance was inserted as the covariate in vancomycin clearance. This final value is similar to two previous studies using general Japanese populations (i.e., 38.5% [15] and 37.5% [6]) but larger than other two specific-type-based studies [4, 16]. Study in critically ill patients [4] and ECMO-supported patients [16] demonstrated interindividual variability of 29.2% and 25%, respectively. Interindividual variability for *V*_1_ in the present study (20.93%) was lower than other two studies (i.e., 25% [16] and 36.4% [4]). However, study from Yamamoto et al. [6] displayed the decreasing of interindividual variability of *V*_1_ from 18.2% to 10.1% when type of infectious disease was taking into account. Other two parameters without covariate (*Q* and *V*_2_) in the present study had higher values of interindividual variabilities (39.50% and 57.27% for interindividual variability for *Q* and *V*_2_, resp.). These results suggested that interindividual variabilities could be affected by other variables that were not assessed in the present study.

The magnitude of variability in drug concentrations observed over time within an individual (intraindividual variability) in the present study was 4.51 mg/L. On the basis of average concentration in this study, which was 17.13 mg/L, this equivalent to an average error of 26.32%. This value was higher than all four previous studies using similar approach with the present study, which were 23.7% [15], 13.2% [6], 23.9% [4], and 12.1% ± 2.1 mg/L [16]. Since the assay error was contributed only less than 5%, other factors might attribute to this result. For fixed effect parameters (*θ*_*n*_), the degree of imprecision ranged from 4.98% to 28.34%. Furthermore, imprecision of estimating interindividual variability of parameter of *V*_1_ (117.00%) and *Q* (158.00%) which represented distribution phase was higher than values for other two parameters (i.e., CL of 22.9% and *V*_2_ of 67.68%). Since most samples were drawn after distribution phase was completed, the poor precision was expected. Additionally, in the case of dialysis patients (accounted as 34.43% of total population in modeling group), rebound phenomenon might be occurred as sharp decreasing of vancomycin concentration during haemodialysis session followed by increasing concentration at the end of hemodialysis session of 3–6 hours [22]. Drawing the level during these periods could mislead the result of the measurement. 

Predictive performance in validation step was carried out by applying the final model to the validation group. The result was not biased and precise, since zero values were included in the value of ME (representative of bias) and RMSE (representative of precision) in the 95% CI. Additionally, the negative value of ME, indicated that the model under predicted vancomycin concentration. 

A scatter plot of predicted concentrations versus weighted residuals shown the potential outliers, which are the points outside the ±3 unit range (Figure 5). In this study, when the data of the covariate in particular day was missing, the other day data was used. This practice could lead to a mistake when the true value was significantly different from replacing value. The discrepancy between the times (of drug administration and drawing the samples) happened and was actually recorded, and also it could not be ignored as the source of error. 

**Figure 6:**
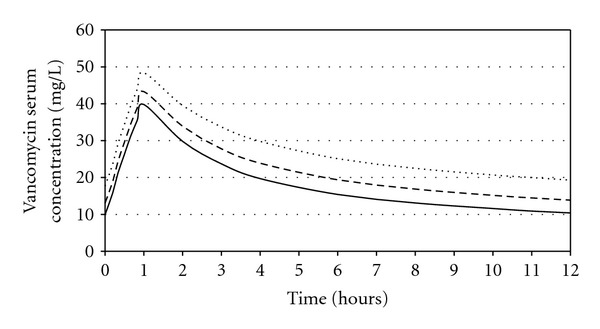
Simulation of steady-state vancomycin serum concentration profile in the 50-year-old patient using dosage regimen of 1 gram/12 hours.

The result from the present study could be used as basic information in dosage regimen calculation for patients with similar characteristics as the present study. However, caution could be drawn in any conditions which were not accommodated by the present study. For instance, since there was no body weight included in the model, obese patients might not be represented by the present model. Simulation using the result from the present study was conducted to determine vancomycin serum concentration when a dosage regimen was applied to particular patient. Time versus steady-state vancomycin serum concentration curves in three different values of creatinine clearance in the 50-year-old patient were created (Figure 6). The widely used dosage regimen of vancomycin in normal renal function (i.e., 1 g every 12 hours) was applied. On the basis of recent guideline [23], vancomycin trough concentration above 10 mg/L was recommended to avoid development of resistance. Additionally, vancomycin trough concentration of 15–20 mg/L was desired to treat complicated infections (endocarditis, osteomyelitis, meningitis, and hospital-acquired pneumonia). In this simulation, if the patient had complicated infection, the dose of 1 g/12 hours seems to be suitable for the patients with CL_Cr_ of 60 mL/min and 80 mL/min. However, in similar circumstances, more aggressive dose might be needed for the patients with CL_Cr_ of 100 mL/min. 

In conclusion, the pharmacokinetic parameters of vancomycin in Thai patients and factors influencing the variability of these pharmacokinetic parameters were established. Furthermore, the inter- and intraindividual variabilities of the pharmacokinetic parameters were determined. The model could be used to obtain the specific patient's pharmacokinetic parameters to create vancomycin dosage regimen in the type of patient similar with the present study.

## Figures and Tables

**Table 1:** Summary of demographic and clinical characteristics of the patients in the modeling group and the validation group.

Characteristics	Modeling group	Validation group	*P* value^a^
Number of patients	212	34	ND^b^
Total number of vancomycin levels	391	68	ND^b^
Average number of vancomycin level per patient	1.84	2.00	ND^b^
Sex distribution	Male: 112 (52.83%); Female: 100 (47.17%)	Male: 12 (35.29%); Female: 22 (64.71%)	0.058
Age, in years (mean ± SD)	66.62 ± 18.38	70.24 ± 18.61	0.288
Total body weight, in kg (mean ± SD)	57.64 ± 11.62	55.81 ± 12.20	0.201
Serum creatinine, in mg/dL (mean ± SD)	3.12 ± 2.92	2.70 ± 2.81	0.421
Blood urea nitrogen, in mg/dL (mean ± SD)	40.25 ± 25.15	46.41 ± 40.06	0.410
Creatinine clearance by Cockcroft-Gault equation, in mL/min (mean ± SD)	35.07 ± 29.83	35.50 ± 30.61	0.939
Creatinine clearance by MDRD4 equation, in mL/min/1.73 m^2^ (mean ± SD)	44.08 ± 36.71	45.36 ± 36.43	0.85
Albumin, in g/L (mean ± SD)	25.93 ± 7.31	28.79 ± 7.53	0.047*
AST, in U/L (mean ± SD)	87.08 ± 177.71	346.52 ± 1620.11	0.396
ALT, in U/L (mean ± SD)	83.27 ± 157.93	156.17 ± 579.60	0.506
Total bilirubin, in mg/dL (mean ± SD)	3.22 ± 7.39	1.85 ± 4.04	0.329
Direct bilirubin, in mg/dL (mean ± SD)	2.38 ± 5.94	1.15 ± 2.90	0.074
Ascites (yes/no/unknown)	43/169/0	5/26/3	0.587
Concomittant medication (yes/no/unknown)			
Aminoglycosides	27/185/0	1/30/3	0.121
Amphotericin B	12/200/0	0/31/3	0.174
NSAIDs	20/192/0	3/27/4	0.921
Dopamine	33/179/0	2/28/4	0.195
Dobutamine	9/203/0	0/30/4	0.250
Adrenaline/noradrenaline	24/188/0	2/28/4	0.441
Furosemide	77/135/0	15/15/4	0.149

^a^Differences between the modeling and validation group were considered to reach statistical significance when *P* < 0.05; ^b^Not determined; *statistically significant.

**Table 2:** Population pharmacokinetic parameters of vancomycin in Thai patients estimated from final model.

Parameter	Mean estimate	Estimation error^a^ (%)
Fixed parameters		
CL (L/h) = *θ*_1_ × CL_Cr_ (mL/min)		
*θ*_1_	0.044	22.34
*V*_1_ (L) = *θ*_2_ × Age (years old)		
*θ*_2_	0.542	16.00
*Q* (L/h) = *θ*_3_		
*θ*_3_	6.950	45.17
*V*_2_ (L) = *θ*_4_		
*θ*_4_	44.200	19.58

Interindividual variability		
*ω*_CL_ (%)	35.78	20.00
*ω*_*V*_1__ (%)	20.93	120.00
*ω*_*Q*_ (%)	39.50	346.67
*ω*_*V*_2__ (%)	57.27	75.47

Intraindividual variability		
*σ* (mg/L)	4.51	38.07

^a^Expressed as a coefficient of variation.

CL, vancomycin clearance; *V*_1_, volume of the central compartment of vancomycin; *Q*, vancomycin intercompartmental clearance; *V*_2_, volume of the peripheral compartment of vancomycin; CL_Cr_, creatinine clearance by Cockcroft-Gault equation; *ω*, interindividual variability related to PK parameter; *σ*, intraindividual variability.
